# Dysfunction of Shh signaling activates autophagy to inhibit trophoblast motility in recurrent miscarriage

**DOI:** 10.1038/s12276-020-00530-6

**Published:** 2021-01-04

**Authors:** Yibin Pan, Lili Yan, Qiaoqiao Chen, Cheng Wei, Yongdong Dai, Xiaomei Tong, Haiyan Zhu, Meifei Lu, Yanling Zhang, Xiaoying Jin, Tai Zhang, Xiaona Lin, Feng Zhou, Songying Zhang

**Affiliations:** 1 Assisted Reproduction Unit, Department of Obstetrics and Gynecology, Sir Run Run Shaw Hospital, Zhejiang University School of Medicine, Hangzhou, China; 2 Key Laboratory of Reproductive Dysfunction Management of Zhejiang Province, Hangzhou, China; 3 Beilun District Hospital of Traditional Chinese Medicine, Ningbo City, Zhejiang China; 4 Department of Pharmacy, The Children’s Hospital, Zhejiang University School of Medicine, Hangzhou, China

**Keywords:** Embryology, Endocrine reproductive disorders, Autophagosomes

## Abstract

In early pregnancy, the placenta anchors the conceptus and supports embryonic development and survival. This study aimed to investigate the underlying functions of Shh signaling in recurrent miscarriage (RM), a serious disorder of pregnancy. In the present study, Shh and Gli2 were mainly observed in cytotrophoblasts (CTBs), Ptch was mainly observed in syncytiotrophoblasts (STBs), and Smo and Gli3 were expressed in both CTBs and STBs. Shh signaling was significantly impaired in human placenta tissue from recurrent miscarriage patients compared to that of gestational age-matched normal controls. VEGF-A and CD31 protein levels were also significantly decreased in recurrent miscarriage patients. Furthermore, inhibition of Shh signaling impaired the motility of JAR cells by regulating the expression of Gli2 and Gli3. Intriguingly, inhibition of Shh signaling also triggered autophagy and autolysosome accumulation. Additionally, knockdown of BECN1 reversed Gant61-induced motility inhibition. In conclusion, our results showed that dysfunction of Shh signaling activated autophagy to inhibit trophoblast motility, which suggests the Shh pathway and autophagy as potential targets for RM therapy.

## Recurrent miscarriage: Malfunctioning molecular signals

Impairments in a signaling process in embryological development process are linked to problems in early pregnancy that can cause recurrent miscarriages, an insight that could suggest new targets for preventive treatment. The signaling process, named after two of its key proteins, Shh and Gli, controls sequences of gene activation and repression that are vital for the early development of the placenta and embryo. Songying Zhang and colleagues at Zhejiang University School of Medicine, Hangzhou, China, examined placental tissues from normal but aborted pregnancies and from patients with recurrent miscarriage. In the latter group, they found that dysfunctional Shh/Gli signaling impaired the activity of cells needed to form the placenta, and reduced blood vessel formation. The dysfunction also increased the destruction of cell components in these cells.

## Introduction

In mammals, after zygote division and development into blastocysts, the outer cells of blastocysts become polarized and then differentiate, first into trophoblasts and then further to form the placenta. The inner cells of blastocysts divide to form the inner cell masses, which further differentiate to form the embryo proper. The human placenta serves as the feto-maternal material exchange barrier and protective shield to give rise to fetal development. Physiologically, trophoblast progenitor cells differentiate into cytotrophoblasts (CTBs). CTBs either differentiate into invasive lineages to yield extravillous trophoblasts (EVTs) or undergo cell fusion to yield syncytiotrophoblasts (STBs)^1^. EVTs are further subdivided according to their anatomical location and degree of differentiation^2^. Those that invade the decidualized endometrium are called interstitial EVTs, and they have a pivotal role in implantation^3^. Those that invade and remodel the spiral arteries are called endovascular EVTs. Other subtypes have also been detected in uterine glands, veins, and the lymphatic system^4,5^. The integrated proliferation and differentiation of all these trophoblast lineages are essential for normal placental development. Aberrant trophoblast development is usually associated with severe pregnancy complications, including miscarriage, preeclampsia, and intrauterine growth restriction^6–8^.

Recurrent miscarriage is a common pregnancy-related complication and is defined as more than two consecutive spontaneous miscarriages; this problem affects more than 5% of reproductive-aged women^9^. The pathogenesis of recurrent miscarriage is complex and may include hereditary factors, hormone disorders, abnormal anatomical conditions of the uterus or cervix, autoimmune disorders, or infections of the uterus or cervix^10–15^. However, the signaling networks involved in recurrent miscarriage are not clearly understood. A recent study demonstrated that early miscarriage occurs mainly due to cytotrophoblast dysfunction, including abnormal trophoblastic proliferation and reduction of endovascular EVT penetration^16^. Low expression of MFN2 causes dysfunction of trophoblast cells, including aberrant activation of autophagy and mitochondrial damage, which in turn result in early unexplained miscarriage^17^. The activities of ERK and AKT signaling were found to be significantly suppressed in recurrent miscarriage patients, yet the downstream effectors remain unknown^18^. The miR‐27a‐3p/USP25 axis regulates the trophoblast EMT process and might become a biomarker for recurrent miscarriage^19^. The expression of peroxiredoxin2, which regulates trophoblast proliferation and apoptosis, was decreased in recurrent miscarriage^20^. Enhancer of zeste homolog 2 (EZH2), known as an epigenetic factor that promotes trophoblast invasion, was also downregulated in recurrent miscarriage^21^. These findings indicate that the initiation and progression of recurrent miscarriage is a complex process that involves multiple signaling pathways.

Sonic hedgehog (Shh), together with Indian hedgehog (Ihh) and Desert hedgehog (Dhh), is a ligand of the Hedgehog (Hh) signaling pathway. This pathway has pivotal roles in regulating cell proliferation, cell differentiation, organogenesis, and development and is even involved in tumorigenesis and progression^22,23^. A previous review of Hh signaling indicated that this pathway has important roles in regulating hematopoiesis, vasculogenesis, and angiogenesis during embryogenesis and development^24,25^. Moreover, Shh might engage in crosstalk with the TGF/SMAD signaling pathway to promote G-CSF mobilized human CD34^+^ cell migration, proliferation, and then differentiation into vascular cells during embryonic vascular development^26^. Our previous study found that Shh, together with Gli2/3, was required for the proper development of the placenta and maintenance of pregnancy^27^. In addition, we also found that Hh signaling through GLI1/2 promoted the EMT process in human trophoblast cells via transcriptional suppression of the CDH1 gene^28^. However, the relationship between Shh and recurrent miscarriage, as well as the underlying functions of Shh in recurrent miscarriage, have not been investigated.

Autophagy is a lysosomal degradative process that contributes to developmental processes and maintenance of homeostasis^29–31^. Thus far, the roles of Hh signaling in regulating autophagy remain elusive. The inhibition of Hh signaling induces autophagy in *Drosophila*, HeLa cells, and human hepatocellular carcinoma cells^32,33^. However, Hh signaling acts as a positive regulator of autophagy in vascular smooth cells and hippocampal neurons^34,35^. Additionally, the combined inhibition of Hh signaling and autophagy was found to overcome the chemoresistance of chronic myeloid leukemia, and this phenomenon was associated with PARP cleavage, CASP3 and CASP9 cleavage, and the BCR-ABL oncoprotein^36^. Other studies have shown that autophagy is highly activated in EVTs and placentas, and its disruption is associated with preeclampsia and intrauterine growth restriction^37–41^. However, the effect of autophagy on recurrent miscarriage remains largely elusive.

In the present study, we investigated the potential role of Shh/Gli signaling and autophagy in recurrent miscarriage. Our results showed that Shh signaling was attenuated in the placenta of recurrent miscarriage patients, and dysfunction of Shh/Gli impaired trophoblast migration and angiogenesis. Moreover, inhibition of Shh signaling enhanced autophagy, while inhibition of autophagy reversed Gant-6-induced inhibition of trophoblast motility. These findings have important implications for the pathological role of Shh signaling and autophagy in recurrent miscarriage and might provide potential targets for recurrent miscarriage therapy.

## Materials and methods

### Preparation of placental tissues

Placental villi tissues were isolated from healthy pregnant women who were undergoing elective abortion (*n* = 10). The women were 27–40 years old, and the abortions were performed at 49–82 days of gestation. Recurrent miscarriage patients (*n* = 10) were 24–37 years old (mean age 32.2 ± 5.45 years) and experienced spontaneous abortion at 33–66 days of gestation (mean gestational age 44.2 ± 12.83 days). None of them had any risk factors, such as genetic abnormalities (maternal or paternal), uterine malformation, thyroid dysfunction, or anti-phospholipid antibody syndrome. All placental tissues were stored at −80 °C for further analysis or stored in 4% formaldehyde at room temperature overnight for immunohistochemistry analysis. The study protocol was approved by the ethics committee of the Sir Run Run Shaw Hospital, Zhejiang University School of Medicine.

### First-trimester villous explant and cell line culture

Placental villi were acquired from first-trimester tissues (6–9 weeks of gestation), as described previously^42^. In brief, 8–10 explants (2–3 mm) from the tips of the placental villi were dissected and explanted in 24-well culture dishes precoated with 5 mg/ml Matrigel for 30 min at 37 °C. After 2–4 h for adherence, tissues were carefully covered with 0.5 ml serum-free DMEM/F12 medium and incubated for 24 h. The explants with successful attachment were selected for the treatment and photographed. The outgrowth of trophoblasts was measured with ImageJ software from the National Institutes of Health (http://rsb.info.nih.gov/ij/download.html).

JAR cells (a gift from Dr Hai-Tao Pan, Shaoxing Women, and Children’s Hospital) were used in this study. The complete growth medium for this cell line was basic RPMI-1640 medium supplemented with 10% fetal bovine serum, 100 U/ml penicillin, and 100 μg/ml streptomycin (Gibco by Life Technologies, USA). The cells were incubated at 37 °C with 5% CO_2_.

### Chemicals, reagents, and oligonucleotides

Cyclopamine (Tocris, 1623, USA), recombinant human Shh (R&D, 1845-SH), chloroquine (Sigma, C6628), GANT61 (Selleck Chemicals, S8075), Lipofectamine 3000 (Thermo Fisher Scientific, L3000015), Gli2 shRNA1 (5′-GUACCAUUACGAGCCUCAUUC-3′), Gli2 shRNA2 (5′-CAACGCCCCCCACCCGUAC-3′), Gli3 shRNA1 (5′-UUGAAGGUUGCACAAAGGC-3′), Gli3 shRNA2 (5′-AAGAGAUUAAACUGACUUU-3′), BECN1 shRNA1 (5′-GGATGACA GTGAACAGTTA-3′), and BECN1 shRNA2 (5′-CCCGTGGAATGGAATGAGA-3′).

### Real-time PCR

Total RNA was isolated by using TRIzol reagent (Takara, China) according to the manufacturer’s instructions. Total RNA (2 μg) was reverse transcribed using SuperScript III reagent (Life Technologies) according to the manufacturer’s instructions. After the termination of cDNA synthesis, each reaction mixture was diluted with 80 μl Tris–EDTA buffer. Target genes were determined by RT-PCR and quantitative RT-PCR. Primers were used as follows: *PTCH1* Forward 5′-ACCAAGTGATCGTGGAAGCC-3′, Reverse 5′-GTGGGTGATGCCTGGATTCG-3′; *SMO* Forward 5′-TCGAATCGCTACCCTGCTG-3′, Reverse 5′-CAAGCCTCATGGTGC CATCT-3′; *Gli1* Forward 5′-GGGTGCCGGAAGTCATACTC-3′, Reverse 5′-GCTAGGATC TGTATAGCGTTTGG-3′; *Gli2* Forward 5′-CATGGAGCACTACCTCCGTTC-3′, Reverse 5′-CGAGGGTCATCTGGTGGTAAT-3′; *Gli3* Forward 5′-TGGTTACATGGAGCCCCACTA-3′, Reverse 5′-GAATCGGAGATGGATCGTAATGG-3′; *GAPDH* Forward 5′-AGCCTCAAGAT CATCAGC-3′, Reverse 5′-GAGTCCTTCCACGATACC-3′. GAPDH served as the housekeeping gene. Gene relative expression was calculated by the 2 − ΔΔCt method^43^.

### Cell Counting Kit-8 assay

JAR cells were seeded into 96-well plates at 1000 cells/well and cultured for 24 h. Then, the culture medium was replaced with a fresh complete growth medium plus a final concentration of 5 μM cyclopamine (Tocris, 1623, USA) or 0.5 μg/ml recombinant human Shh (R&D, 1845-SH). Complete growth medium plus an equal volume of alcohol was used as a negative control. The maintenance medium was refreshed every two days with the same formulation as in the previous treatment. At the indicated time points, 10 μl Cell Counting Kit-8 (Dojindo, Japan) solution was added to each well and then incubated for 1 h. The absorbance, indicating cell viability, was measured by SpectraMax M5 (Molecular Devices, USA) according to the manufacturer’s instructions.

### Flow cytometry for the apoptosis assay

JAR cells (5 × 10^5^ cells) were seeded into 60 mm disks and cultured for 24 h. The culture medium was then replaced with a fresh complete growth medium plus a final concentration of 5 μM cyclopamine or 0.5 μg/ml recombinant human Shh and cultured for the next 24 h. Complete growth medium plus an equal volume of alcohol was used as a negative control. At the endpoint of treatment, cells were gently detached by TrypLE™ Express Enzyme (1×) (Gibco by Life Technologies, USA). Cells were washed and resuspended in 100 μl binding buffer, and 5 μl PE-conjugated Annexin V and 5 μl 7-AAD were added immediately thereafter and incubated for 15 min in the dark at room temperature. After incubation, 400 μl of binding buffer was added to all samples, and apoptosis was immediately quantified by using a FACSCanto II flow cytometer (BD Biosciences, USA) according to the manufacturer’s instructions.

### Cell migration and invasion assay

For the migration assay, JAR cells were resuspended in 100 μl basic RPMI-1640 medium and seeded into 24-well transwell polystyrene plates (COSTAR, USA) at 3 × 10^5^ cells/well. For the invasion assay, JAR cells were seeded into 24-well transwell polystyrene plates precoated with Matrigel (BD, USA). After transfection with various shRNAs (scramble shRNA, shGli2, shGli3 or shBeclin1), transfected cells were divided into three groups, and 100 μl basic RPMI-1640 medium plus a final concentration of 5 μM cyclopamine or 0.5 μg/ml recombinant human Shh was added to each well. Basic RPMI-1640 medium plus an equal volume of alcohol was used as a negative control. The lower chambers were loaded with 600 μl fresh complete growth medium in each well. After culturing for 24 h, cells on the inner insert were wiped away with cotton sticks, and migrated cells were fixed with 75% alcohol for 10 min at room temperature and then stained with 0.1% crystal violet. After washing with PBS twice, migrated cells were imaged under a microscope, and each image was analyzed by ImageJ software.

### Western blot

Western blotting was performed as described previously^27^. Briefly, tissue or cell samples were lysed on ice in RIPA lysis buffer containing 1% PMSF (Beyotime, China). Total protein samples (40 μg) were separated by SDS-PAGE and then transferred onto a PVDF membrane. Primary antibodies were used as follows: anti-E-cadherin (Cell Signaling, 3195), anti-VEGF-A (Abcam, ab46154), anti-Sonic Hedgehog (Abcam, ab53281), anti-Gli2 (Abcam, ab2605556), anti-Gli3 (Abcam, ab6050), anti-p-AKT T308 (Cell Signaling, 4056), anti-p-AKT S473 (Cell Signaling, 4046), anti-pan-AKT (Cell Signaling, 4691), Beclin1 (Cell Signaling, 3495), and anti-LC3B (Sigma-Aldrich, L7543). GAPDH (Proteintech, 600004-1-Ig) or β-actin (Cell Signaling, 4970) was used as an internal standard. Immunofluorescent anti-rabbit and anti-mouse secondary antibodies were purchased from LI-COR Bioscience (Lincoln, USA), and the signals were visualized with an Odyssey Infrared Imaging System (Lincoln, USA). Anti-rabbit-HRP and anti-mouse-HRP secondary antibodies were purchased from Beyotime (Shanghai, China). After ECL exposure, images were captured by an Amersham Imager 600 (GE, USA). ImageJ software was used to quantify the immunoreactive bands.

### Immunohistochemistry

For paraffin-embedded sections, samples were normally deparaffinized and rehydrated in xylene and a graded series of ethanol; for antigen retrieval, samples were boiled in a citric acid buffer (pH 6.0) and then incubated with 3% H_2_O_2_ to suppress endogenous peroxidase activity. Samples were further blocked in 10% normal serum with 1% BSA in TBS for 1 h at room temperature and then incubated with primary antibodies overnight at 4 °C. Then, the sections were stained with HRP-conjugated secondary antibodies for 30 min, followed by incubation with diaminobenzidine (DAB) solution and counterstaining with hematoxylin. Primary antibodies used in immunohistochemistry were as follows: anti-Sonic Hedgehog (Abcam, ab53281), anti-Cytokeratin 7 (Gene Tech, GM701802), anti-VEGF-A (Abcam, ab51745), and anti-CD31 (Abcam, ab134168).

For frozen sections or cells grown on glass coverslips, samples were incubated in 0.3% Triton X-100 for 10 min, blocked in 5% goat serum for 1 h at room temperature, and incubated with primary antibodies overnight at 4 °C. Then, the sections were incubated with fluorescent secondary antibodies for 1 h at room temperature in the dark and mounted in a drop of mounting medium containing DAPI. The images were captured by an Olympus BX53 fluorescence microscope and then analyzed with ImageJ software. The primary antibodies used for this experiment were as follows: anti-Sonic Hedgehog (Abcam, ab53281), anti-Ptc1 (Abcam, ab109096), anti-Smo (Abcam, ab72130), anti-Gli1 (Abcam, ab92611), anti-Gli2 (Abcam, ab26056), anti-Gli3 (Abcam, ab6050), anti-Cytokeratin 7 (CK7), anti-11 β-HSD2 (Santa Cruz, sc-365529), anti-LC3B (Sigma-Aldrich, L7543), and anti-LAMP1 (Abcam, ab25630).

### RNA-seq

JAR cells were seeded into 10 cm cell culture dishes and treated with or without 5 μM cyclopamine (Biomol International, USA) for 24 h. Total RNA from each group was extracted with TRIzol (Invitrogen) reagent. Then, samples were processed, and RNA-seq was performed by the Annoroad Gene Technology company (Beijing, China). The analysis platform was the Illumina HiSeq 2500 platform with a 101-bp paired-end sequencing strategy. The complete array data set has been deposited into the Gene Expression Omnibus (GEO accession number: GSE130367). Gene set enrichment analysis (GSEA, http://gsea.org/) was performed to identify the functions of differentially expressed mRNAs and associated enriched pathways.

### Analysis of GFP-mCherry-LC3 puncta

For autophagosome maturation assays, JAR cells were transfected with the GFP-mCherry-LC3 adenovirus vector (5 × 10^9^ pfu/ml) for more than 24 h and treated with or without the Hh antagonist chloroquine (CQ) for 24 h. GFP fluorescence is diminished in acidic autolysosomes, whereas mCherry fluorescence remains stable. Images of GFP-mCherry-LC3 puncta were captured by an Olympus BX53 fluorescence microscope, and ImageJ software was then used for quantification as described^44^.

### Statistical analysis

All numerical data are expressed as the mean ± S.E.M and were analyzed by Student’s *t*-test or one-way ANOVA (SPSS 13.0J software; SPSS, Inc., Chicago, IL, USA). Statistical significance was assessed at *P* < 0.05. Experiments were performed independently in triplicate, and the results were qualitatively identical. Representative experiments are shown.

## Results

### Shh signaling was attenuated in recurrent miscarriage

**Fig. 1:**
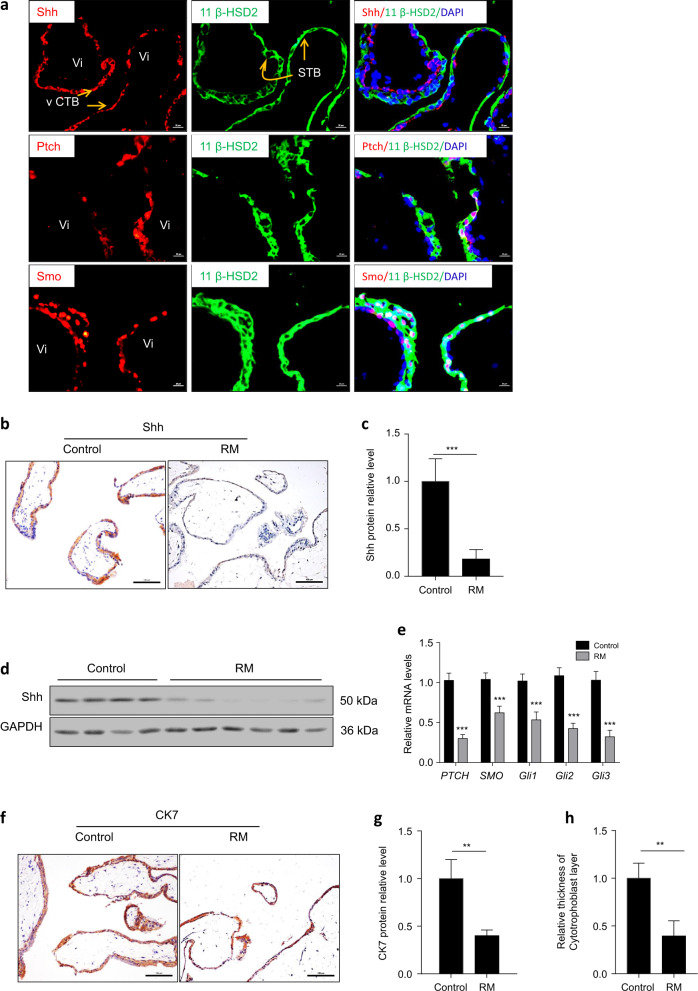
Shh signaling was impaired in recurrent miscarriage patients’ villous tissues. **a** Double immunofluorescence staining of Shh, Ptch, Smo (red), and 11 β-HSD2 (green) in first-trimester villous tissue from healthy controls, nuclei were counterstained with DAPI (blue). Scale bars, 20 μm. **b** Expression of Shh protein in control group and recurrent miscarriage (RM) group were detected by immunohistochemistry. Scale bars, 100 μm. **c** Expression of Shh from panel **b** was analyzed and quantified by Image J software. **d** Expression of Shh protein in control group and recurrent miscarriage group were detected by western blot. **e** RNA expression of *PTCH*, *SMO*, *Gli1/2/3* in control group and recurrent miscarriage group were detected by real-time PCR. **f** CK7 staining in control group and recurrent miscarriage group were detected by immunohistochemistry. Scale bars, 100 μm. **g** Expression of CK7 from panel **f** was analyzed and quantified by Image J software. **h** Relative thickness of cytotrophoblast layer from **f** was analyzed and quantified by Image J software. *p < 0.05, **p < 0.01, ***p < 0.001.

We first investigated the expression and cellular localization of Shh signaling core members in villous tissue from first-trimester placentas. 11-β-HSD2 serves as a marker of the STB layer and has important roles in fetal development^45^. Our results showed that both Shh and Smo were expressed in the STB layer and CTB layer, but Shh was mainly located in the CTB layer. Ptch was mainly located in the STB layer (Fig. 1a). Compared to normal human placental villi (the healthy control), Shh was decreased in villous tissues from recurrent miscarriage patients (Fig. 1b, c). This result was also confirmed by immunoblotting of Shh in protein extracts from villous tissues from healthy controls and recurrent miscarriage patients (Fig. 1d). Moreover, RNA expression of *PTCH, SMO*, and *Gli1/2/3* was significantly downregulated in recurrent miscarriage patients compared to the controls (Fig. 1e). In addition, CK7, a marker of trophoblasts, was decreased in villous tissues from recurrent miscarriage patients (Fig. 1f, g), and the thickness of the CTB layer was significantly decreased in recurrent miscarriage patients compared to healthy controls (Fig. 1g, h). Thus, Shh signaling was impaired in the villous tissues of recurrent miscarriage patients.

### Attenuating Shh signaling inhibited trophoblast motility and placental angiogenesis

**Fig. 2:**
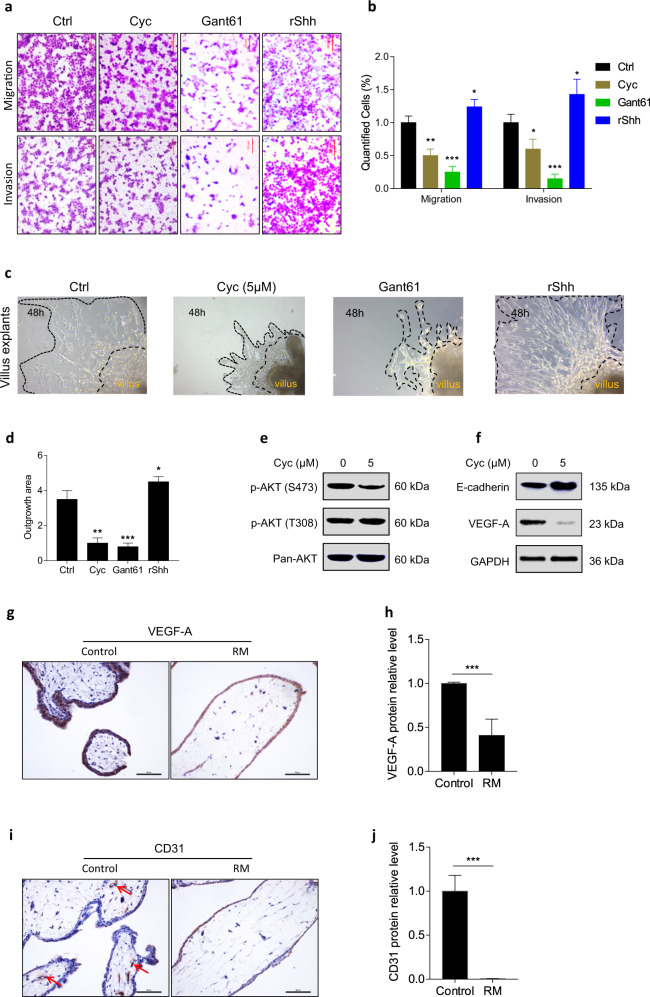
Attenuating Shh signaling inhibited trophoblast motility and placental angiogenesis. **a** The effect of cyclopamine, Gant61 or recombinant Shh 10.1038/s12276-020-00530-6 on regulating migration and invasion of JAR cells. Scale bars, 200 μm. **b** Migrated or invaded cells from panel **a** were quantified by Image J software. **c** The effect of cyclopamine, Gant61 orrecombinant Shh on regulating motility of extravillous trophoblasts in first-trimester villous. Scale bars, 500 μm. **d** Outgrowth area from panel **c** was quantified by Image J software. **e** After with or without cyclopamine treatment, expression of p-AKT S473, p-AKT T308, and pan-AKT proteins were detected by western blot. **f** After with or without cyclopamine treatment, expression of E-Cadherin, VEGF-A, and GAPDH proteins were detected by western blot. **g** VEGF-Astaining in control group and recurrent miscarriage group were detected by immunohistochemistry. Scale bars, 100 μm. **h** Expression of VEGF-A from panel **g** was analyzed and quantified by Image J software. **i** CD31 staining in control group and recurrent miscarriage group were detected by immunohistochemistry. Scale bars, 100 μm. **j** Expression of CD31 from panel **i** was analyzed and quantified by Image J software. *p < 0.05, **p < 0.01, ***p < 0.001.

To study the role of Shh signaling in trophoblast motility, Matrigel cell invasion, and transwell cell migration assays were performed. Our results showed that compared to the control treatment, administration of the Smo antagonist cyclopamine (Cyc) or Gli1/2 antagonist Gant61 significantly decreased migration and invasion in JAR cells, while treatment with recombinant Shh significantly increased migration and invasion (Fig. 2a, b). We then investigated the effect of cyclopamine or recombinant human Shh (rShh) on JAR cell viability. Our results showed that 5 μM cyclopamine 0.5 μg/ml rShh had no effect on JAR cell proliferation or apoptosis compared to the control group (Supplementary Fig. s1). However, cyclopamine and Gant61 significantly decreased, while recombinant Shh significantly increased, the outgrowth of invasive extravillous trophoblasts in first-trimester villous explant cultures seeded on collagen I (Fig. 2c, d). At the molecular level, cyclopamine significantly decreased the phosphorylation of the AKT S473 site but not the AKT T308 site (Fig. 2e). VEGF-A is a crucial factor in vasculogenesis during placental development. Our results further showed that cyclopamine significantly decreased VEGF-A expression and significantly elevated E-cadherin expression in JAR cells (Fig. 2f). Moreover, VEGF-A and CD31 expression were significantly decreased in recurrent miscarriage tissues, while VEGF-A was strongly expressed in trophoblasts and CD31 was strongly expressed in the fetal vessels in villous tissue from healthy controls (Fig. 2g–j). These results indicate that downregulation of trophoblast motility might account for aberrant placental vessel regression in recurrent miscarriage patients.

### Shh signaling regulated the motility of JAR cells via Gli2 and Gli3

**Fig. 3:**
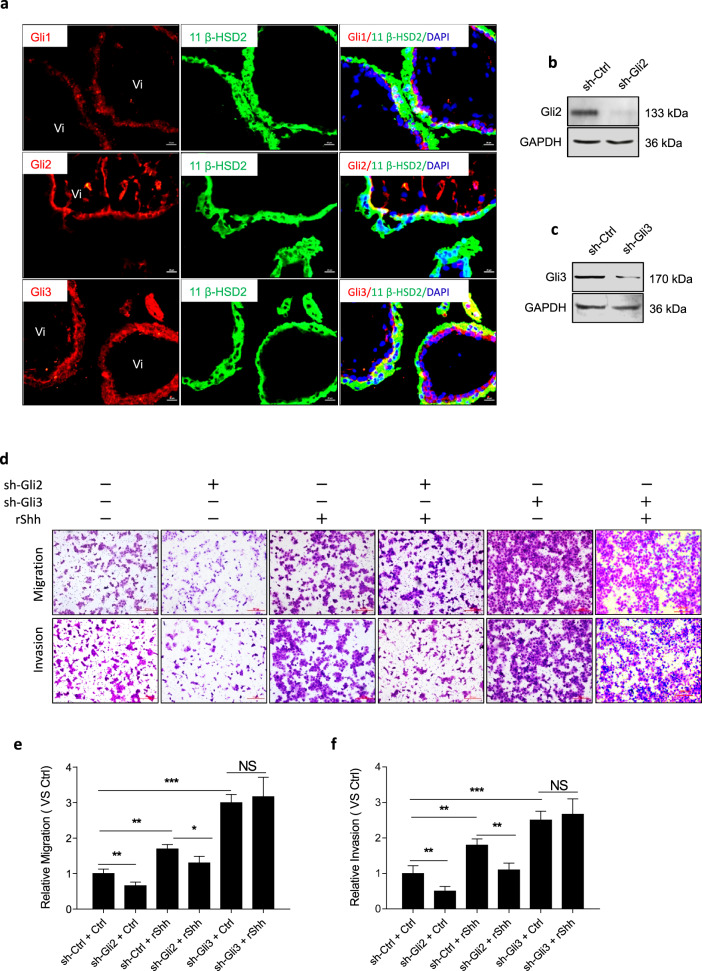
Shh signaling regulated motility of JAR cells via Gli2 and Gli3. **a** Double immunofluorescence staining of Gli1, Gli2, Gli3 (red), and 11 β-HSD2 (green) in normal human first-trimester villous tissues, nuclei were counterstained with DAPI (blue). Scale bars, 20 μm. **b** Immunoblot analysis confirmed the Gli2 knockdown efficiency in sh-Ctrl-JAR cells and sh-Gli2-JAR cells. **c** Immunoblot analysis confirmed the Gli3 knockdown efficiency in sh-Ctrl-JAR cells and sh-Gli3-JAR cells. **d** After indicated treatments, migration and invasion of JAR cells were measured in matrigel cell invasion and transwell cell migration assays. Scale bars, 200 μm. **e** Migrated cell from d was quantified by Image J software, each group was normalized to “sh-Ctrl + Ctrl” group. **f** Invaded cell from panel **d** was quantified by Image J software, each group was normalized to “sh-Ctrl + Ctrl” group. *p < 0.05, **p < 0.01, ***p < 0.001.

To identify factors downstream of Shh signaling that regulate the motility of JAR cells, we first investigated the expression of Gli in normal human first-trimester placental tissues. Our results showed that Gli1 was weakly detected in the STB layer, while Gli2 was strongly expressed in the CTB layer, and Gli3 was strongly expressed in both the STB layer and CTB layer (Fig. 3a). We previously showed that Hh regulates EMT in JEG3 cells through Gli1 and Gli2^46^. Herein, we successfully stably downregulated the expression of Gli2 and Gli3 in JAR cells for further experiments (Fig. 3b, c). Gli2 knockdown significantly inhibited the migration and invasion of JAR cells; moreover, Gli2 knockdown partly impaired recombinant Shh-induced migration and invasion of JAR cells. Gli3 knockdown alone or with recombinant Shh treatment significantly promoted the migration and invasion of JAR cells. However, Gli3 knockdown had no effect on recombinant Shh-induced migration and invasion (Fig. 3d–f).

### Transcriptome profiling of JAR treated with cyclopamine

**Fig. 4:**
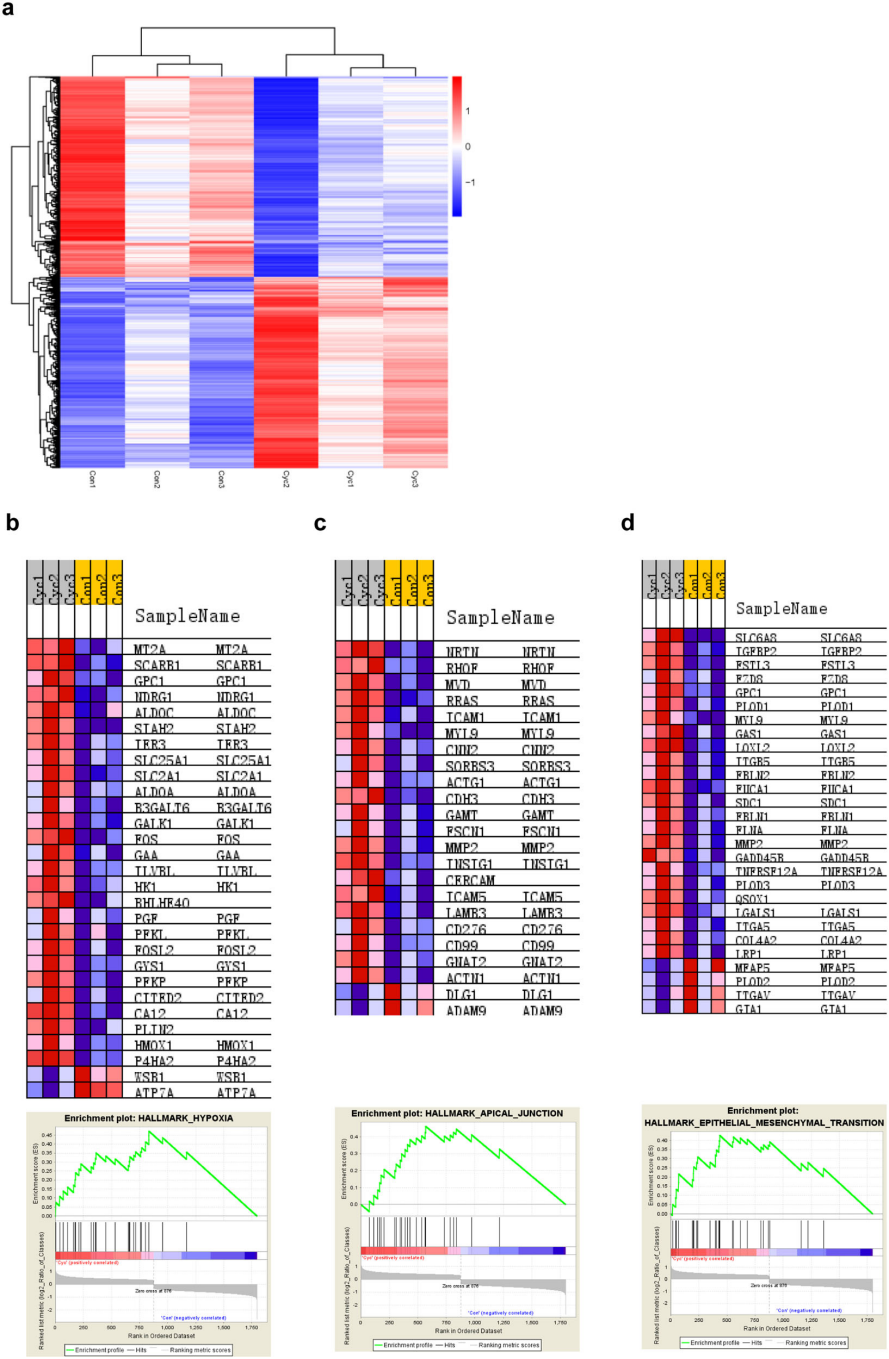
Transcriptome Profiling of JAR treated with Cyclopamine. **a** Heat map depicting hierarchical clustering in JAR cells under 5 μM cyclopamine treatmentfor 24 h. Red represents the high relative expression, while blue represents the low relative expression. **b** Gene Set Enrichment Analysis enriched “Hypoxia” pathway. Enrichment score of 0.47 and a normalized enrichment score of 2.88 (nominal *p*-value ≤ 0.001, false discovery rate ≤ 0.001). **c** Gene Set Enrichment Analysis enriched “Apical Junction” pathway. Enrichment score of 0.46 and an normalized enrichment score of 2.51 (nominal *p*-value ≤ 0.001, false discovery rate ≤ 0.001). **d** Gene Set Enrichment Analysis enriched “EMT” pathway. Enrichment score of 0.43 and a normalized enrichment score of 2.51 (nominal p-value ≤ 0.001, false discovery rate ≤ 0.001).

To screen for additional cyclopamine-regulated target genes in JAR cells, we analyzed the transcriptome profile of JAR cells with or without cyclopamine treatment. RNA-seq data identified 1797 significantly differentially expressed genes (*P* ≤ 0.05, *q* ≤ 0.05, fold change ≥ 2); of these, 876 genes were upregulated, and 921 genes were downregulated (Fig. 4a). Gene set enrichment analysis (GSEA) showed that hypoxia, apical junction, and EMT were the top three most significantly associated pathways, with overall enrichment scores of 0.47, 0.46, and 0.43, respectively, and normalized enrichment scores of 2.88, 2.51, and 2.51, respectively (nominal p-value ≤0.001, false discovery rate {FDR} ≤ 0.001) (Fig. 4b–d).

### Inhibition of Shh signaling induces autophagy and autolysosome accumulation

**Fig. 5:**
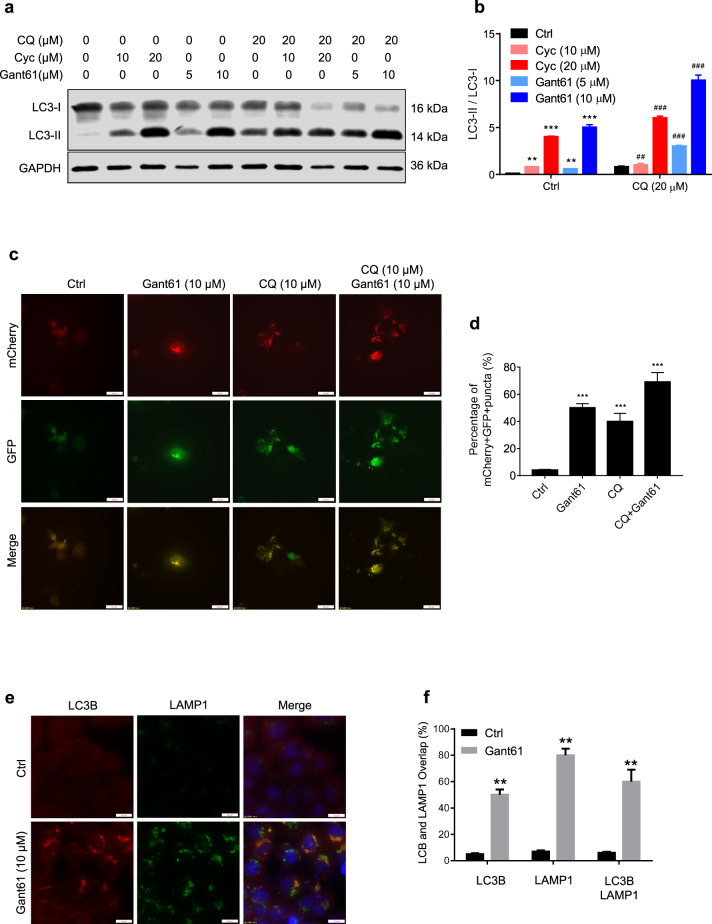
Inhibition of Shh signaling induces autophagy and autolysosome. **a** After treating with or without indicated dose of chloroquine (CQ), cyclopamine and/or Gant61 in JAR cells for 24 h, protein levels of LC3B and GAPDH were measured by western blot assays. **b** Expression of LC3B/GAPDH from panel **a** was quantified by ImageJ software. **c**, **d** JAR cells were infected with GFP-mCherry-LC3 adenovirus vector (5 × 109 pfu/ml) more than 24 h, followed with Gant61 (10 μM) with or without CQ for 24 h, mCherry-positive GFP-negative (mCherry+ GFP−) puncta were captured by microscope and quantified by ImageJ software. Scale bar, 50 μm. **p < 0.01. **e**, **f** After treating with Gant61 (10 μM), colocalization of LC3 and LAMP1 in JAR cells captured by microscope and quantified by Image J software. Scale bars, 20 μm. *p < 0.05, **p < 0.01, ***p < 0.001.

We next investigated the relationship between Shh signaling and autophagy. Our results showed that cyclopamine and Gant61 significantly elevated the ratio of LC3-II/LC3-I protein levels in a dose-dependent manner, and this accumulation was enhanced by treatment with the lysosomal inhibitor chloroquine (CQ) (Fig. 5a, b). Gant61 increased the number of mCherry^+^ GFP^+^ yellow puncta (indicating colocalization of mCherry and GFP) in both CQ-treated and untreated JAR cells (Fig. 5c, d). In addition, the overlap of LC3B and LAMP1 puncta (the indicator for autophagosome–lysosome fusion) was increased in Gant61-treated JAR cells (Fig. 5e, f).

### Downregulation of BECN1 rescued the Gant61-induced inhibition of cell motility in JAR cells

**Fig. 6:**
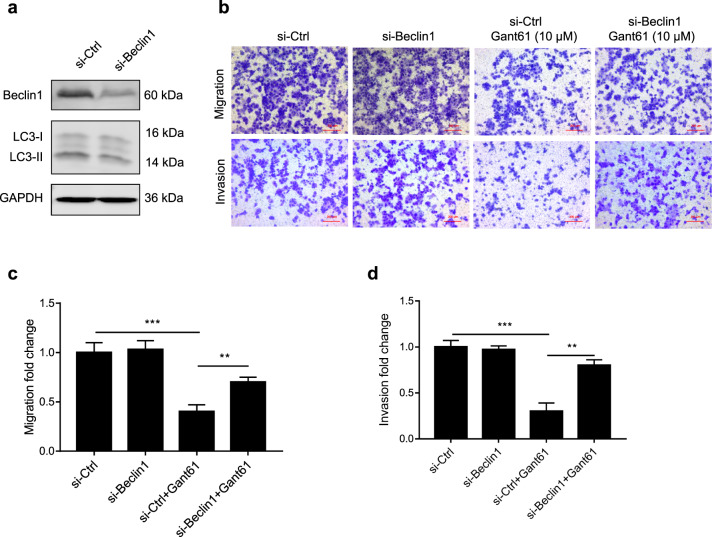
Downregulation BECN1 rescued the Gant61-induced inhibition of cell motility in JAR cells and schematic representation of the role of Shh signaling and autophagy in recurrent miscarriage disease. **a** JAR cells were transiently transfected with Beclin1 siRNAs for 72 h, the protein levels of Beclin1, LC3-I/II, and GAPDH were detected by western blot. **b** After JAR cells were transiently transfected with Beclin1 siRNAs for 48 h, cells were treated with or without 10 μM of Gant61 for another 24 h, migration and invasion of JAR cells were measured by matrigel cell invasion and transwell cell migration assays. Scale bars, 200 μm. **c** Migrated cells from panel **b** wer equantified by Image J software. **d** Invaded cells from **c** were quantified by Image Jsoftware. *p < 0.05, **p < 0.01, ***p < 0.001.

To further investigate the potential role of autophagy in Gant61-induced inhibition of cell motility, we used siRNA to knockdown BECN1, the core autophagy molecule, which is a part of the PI3K complex and regulates the localization of other autophagy proteins to phagophores^47,48^. BECN1 was successfully knocked down in JAR cells, and BECN1 downregulation significantly decreased LC3-II protein levels (Fig. 6a). In addition, downregulation of BECN1 significantly reversed Gant61-induced inhibition of migration and invasion in JAR cells (Fig. 6b–d). These data reveal a critical role for BECN1 in regulating Gant61-induced inhibition of JAR cell motility.

## Discussion

**Fig. 7:**
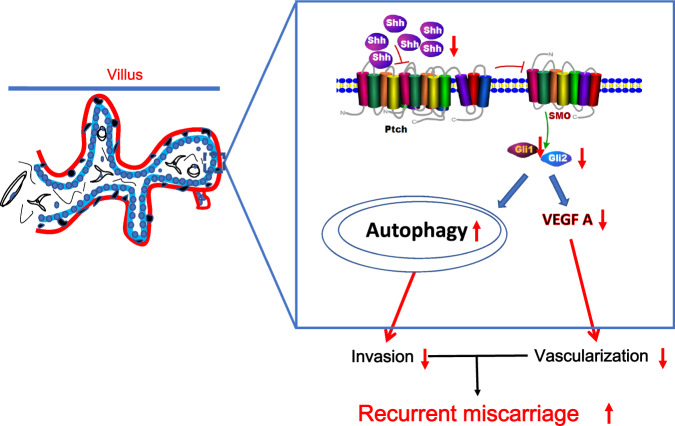
A working model for the role of Shh signaling and autophagy in recurrent miscarriage disease. Downregulation of Shh signaling is related to impaired trophoblast motility and poor placental vascular through autophagy and VEGFA.Shh, autophagy, and VEGFA may provide new insight into the molecular mechanism of recurrent miscarriage, and these signaling pathways may serve as potential therapeutic targets for recurrent miscarriage treatments.

An increasing number of promising studies have improved the prognosis and treatment of unexplained recurrent miscarriage^49,50^. However, the causes and pathophysiology of recurrent miscarriage remain largely elusive. Previous studies have focused on abnormal trophoblast development and placentation in recurrent miscarriage^13,19–21,23,24^. Hh signaling is essential for hematopoiesis, vasculogenesis, and angiogenesis during embryogenesis and development^24,25^. Our previous studies have indicated that Hh signaling has pivotal roles in placental development and pregnancy maintenance^27,28^. In addition, in the clinical context, early miscarriage patients have several common traits, including deficient trophoblastic invasion, decreased CTB layer thickness, and deficient myometrial spiral artery remodeling^16,51,52^. These findings prompted us to further explore the underlying relationship between Shh signaling and recurrent miscarriage. In the present study, our results uncovered crosstalk between Shh signaling and autophagy in regulating trophoblast motility (Fig. 7).

Our previous studies have indicated that Hh signaling is required for EMT in human trophoblast cells, pregnancy maintenance, and placental development^27,28,53–55^. Thus, in the present study, we first investigated the expression of core members of the Shh signaling pathway in healthy human placental villi. Our results showed that the Shh/Ptch/Smo/Gli2/Gli3 signaling axis was preferentially activated in the CTB layer, and Shh signaling was impaired in villous tissue of recurrent miscarriage patients compared to healthy controls. Recently, another study indicated that both previous miscarriages and single nucleotide polymorphisms (SNPs) rs3738880 in Gli2 were associated with anorectal malformations, although the relationship between miscarriages and SNP rs3738880 in Gli2 remains unknown^56^. In addition, conditional deletion of Smo in the mouse uterus impairs implantation and subsequent pregnancy loss^57^.

During placental development, the migration and invasion of trophoblast cells are essential for placental angiogenesis^3,58^. At the molecular level, PAPP-A2 attenuated HTR8/SVneo trophoblast migration and invasion by decreasing the expression of Gli1/2, Snail, Slug, N-cadherin and Vimentin while increasing the expression of E-cadherin and ZO-1^58^. Our previous study also showed that Gli1 induced the expression of Snail, Slug, and Twist1, while Gli2 suppressed the expression of E-cadherin, to promote the migration of JEG3 trophoblast cells^28^. Another study showed that rosiglitazone increased the phosphorylation of AKT to promote endothelial cell migration^59^. In the present study, our results further showed that Shh-Gli2/Gli3 has an important role in JAR migration and invasion. In addition, our results showed that inhibition of Shh signaling decreased AKT phosphorylation while increasing E-cadherin expression in JAR cells. Thus, our results might provide an alternative mechanism by which inactivation of Shh signaling attenuates the migration and invasion of JAR cells by decreasing the phosphorylation of the AKT S473 site while increasing the expression of E-cadherin. Moreover, it has been reported that the inhibition of the Hh pathway induces autophagy by downregulating the AKT-MTOR pathway^60^.

A previous study showed that the luminal epithelial and stromal expression of HIF1α was higher in women with recurrent miscarriage than in fertile controls, suggesting that altered hypoxia and vascularization status may account for the endometrial contribution to recurrent miscarriage^61^. Our results showed that the expression of hypoxia-related genes, such as MT2A, was decreased after cyclopamine treatment. MT2A contributed to proliferation and sprouting as well as to the increased migration of human umbilical vein endothelial cells^62^. It has been shown that the YY1–HOTAIR–MMP2 signaling axis is impaired in recurrent miscarriage^63^. To the best of our knowledge, the relationship between apical junctions and recurrent miscarriage remains largely unknown. Thus, our transcriptome profiling data showed that Shh signaling inhibition was highly correlated with hypoxia, apical junction components, and the EMT pathway, indicating that Shh signaling inhibition would result in aberrant hypoxia and impair the vascularization and mobility of trophoblasts, which would contribute to recurrent miscarriage.

Our RNA-seq results further showed that there was no significant change in autophagy-related genes upon cyclopamine treatment. However, inhibition of Shh signaling promoted autophagosome maturation. Our results also showed that inhibiting autophagy by Beclin1 knockdown reversed Gant61-induced motility inhibition in JAR cells. These results suggest that Shh signaling interacts with autophagy in regulating trophoblast motility.

Accumulating evidence indicates that Hh signaling and VEGF-A are required for placental angiogenesis^24,25,64–66^. In the present study, our results also showed that inhibition of Shh signaling decreased the expression of VEGF-A in JAR cells. Additionally, we found that VEGF-A and CD31 were downregulated in the villous tissue of recurrent miscarriage patients compared to that of healthy controls. These results suggest poor vascular placentation in recurrent miscarriage patients.

In conclusion, we provided an alternative mechanism by which Shh signaling regulates trophoblast cell motility, which subsequently has an important role in placentation and vascularization in recurrent miscarriage patients. In addition, we found that Gant61 could induce autophagy and autolysosome accumulation in trophoblast cells, and Beclin1 responded to Gant61-induced motility inhibition. Taken together, our results indicate an interplay of Shh signaling with autophagy in the human placenta, dysfunctions of which would account for the initiation and progression of recurrent miscarriage. Therefore, restoration of both Shh signaling pathways might be a promising therapeutic strategy to improve vascularization and penetrate maternal spiral arteries to overcome recurrent miscarriage in the future.

## Supplementary information

Figure S1
