# Supplementary material for: Dysfunction of Shh signaling activates autophagy to inhibit trophoblast motility in recurrent miscarriage
Source: Exp Mol Med. 2021 Jan 4;53(1):52–66. doi: 10.1038/s12276-020-00530-6 (PMC8080798; doi:10.1038/s12276-020-00530-6)
Supplement: Supplementary file 1 — Figure S1 [file 12276_2020_530_MOESM1_ESM.docx]

**Fig. s1 Shh signaling does not affect trophoblast viability. a** After treatment of 5 μM cyclopamine (Cyc) or 0.5 μg/ml recombinant human Shh (rShh) in JAR cells, apoptosis was analyzed by flow cytometry. **b** Quantitative apoptosis from **a**. **c** JAR cells were treated with Cyc or 0.5 μg/ml rShh for 24 h, and the cell viability was determined by the CCK-8 assay at 1,2,3,4 and 5 days.
